# Case Report: Cerebral venous thrombosis revealing celiac disease

**DOI:** 10.12688/f1000research.54233.1

**Published:** 2021-07-28

**Authors:** Romdhane Wiem, Arfa Sondess, Chelly Jihene, Jomaa Olfa, Hammami Sonia, Hmida Karima, El Arbi Fatma, Berriche Olfa

**Affiliations:** 1 Department of Endocrinology and Internal Medicine, Tahar Sfar University Hospital, Mahdia, 5100, Tunisia; 2 Faculty of Medicine, University of Monastir, Monastir, 5000, Tunisia; 3 Biochemistry Laboratory, LR12ES05 LR-NAFS Nutrition-Functional Food and Vascular Health, Faculty of Medicine, University of Monastir, Monastir, 5000, Tunisia; 4 Department of Endocrinology and Internal Medicine, Fattouma Bourguiba University Hospital, Monastir, 5000, Tunisia; 5 Department of Radiology, Tahar Sfar University Hospital, Mahdia, 5100, Tunisia

**Keywords:** Celiac disease; venous thromboembolic disease; malabsorption syndrome; Hypercoagulability.

## Abstract

Celiac disease (CD) is an autoimmune enteropathy resulting from intolerance of an individual genetically predisposed to gluten. It has a large clinical polymorphism ranging from a classic digestive clinical presentation due to the malabsorption syndrome to extra-intestinal symptoms. Among the hematologic abnormalities, venous thromboembolic disease (VTE) has been reported, and they are most often located in the abdomen or lower limbs, but the cerebral localization was exceptionally described. We report a case of CD revealed by cerebral thrombophlebitis.

A 44-year-old patient with no medical history and no drug intake, presented with hemiplegia followed by a status epilepticus in a context of apyrexia, initially hospitalized in intensive care. Magnetic imaging resonance displayed a cerebral venous thrombosis of the sigmoid sinus requiring anticoagulant treatment, then transferred to our department for the etiological investigation. On questioning, the patient reported chronic diarrhea and weight loss with no other associated symptoms. The examination revealed an underweight patient with pale conjunctiva, improvement of her deficit symptoms, and no other abnormalities.

Laboratory tests noted biological signs of malabsorption. The thrombophilia assessment revealed a protein C deficiency with a slight increase in anticardiolipin antibodies and anti-Beta 2 glycoprotein 1 antibodies. Immunological tests noted positives anti-transglutaminase and IgA anti-endomysium antibodies. Duodenal biopsy demonstrated villous atrophy. After ruling out the other causes of VTE, the diagnosis of cerebral venous thrombosis secondary to CD was retained.

Early diagnosis and treatment of CD improves the quality-of-life for patients and may spare them various long-term or even fatal complications.

## Introduction

Celiac disease (CD) is an autoimmune enteropathy resulting from intolerance of an individual genetically predisposed to gluten. It affects 0.6–1.0% of the world population. ^1^ It has a large clinical polymorphism ranging from a classic digestive clinical presentation due to the malabsorption syndrome; diarrhea and abdominal pain; to extra-intestinal symptoms. ^2^ It requires lifelong adherence to a gluten-free diet.

Among the hematologic abnormalities, venous thromboembolic disease (VTE) has been reported in the literature, with a 25% higher risk in patients with CD compared with the general population. ^3^ VTE is most often located in the abdomen or lower limbs, but the cerebral localization has been exceptionally described. ^4^

Here, we report a case of CD revealed by cerebral venous thrombosis discovered while exploring a status epilepticus. This presentation has not been reported previously in the literature.

## Case report

**Figure 1.**
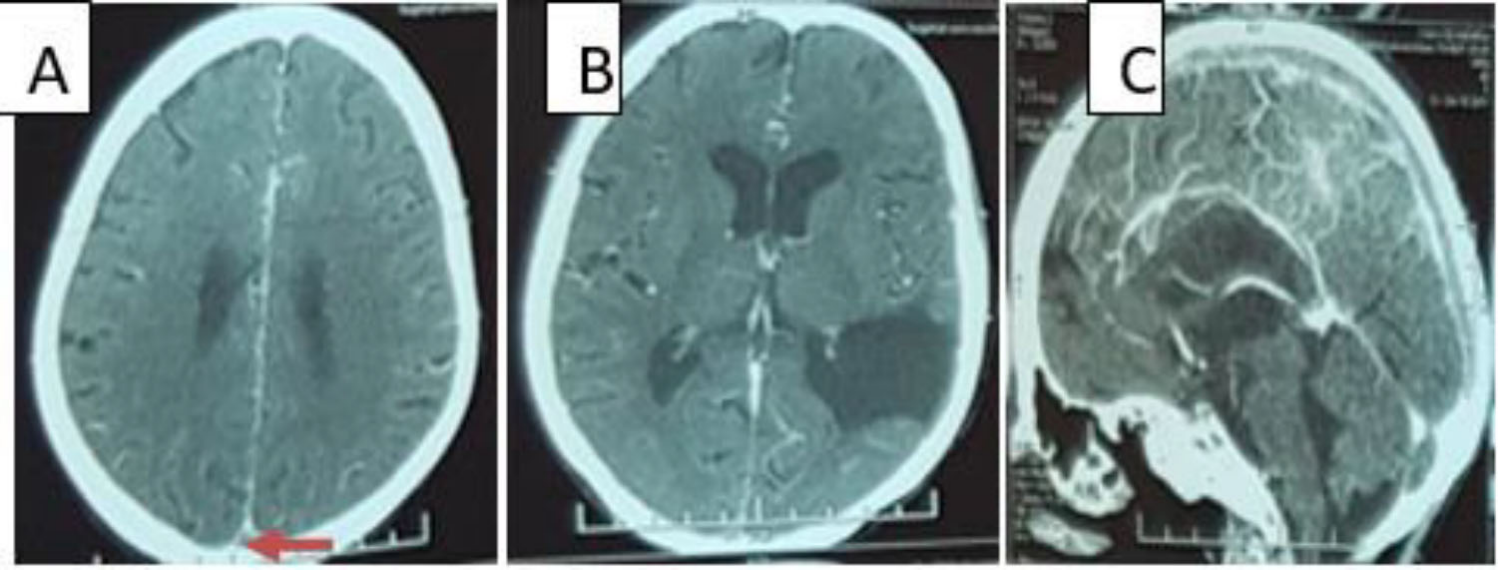
Cerebral CT scan (A, B: axial plane, C: Sagittal plane) showing a venous thrombosis of superior sagittal sinus (A, C) and a left temporoparietal porencephalic cyst (B).

A 44-year-old Tunisian female patient, housewife, with no medical history and no drug intake, presented with hemiplegia followed by a status epilepticus in a context of apyrexia, initially hospitalized in intensive care. Neuroimaging displayed a cerebral venous thrombosis of the superior sagittal sinus ( Figure 1) requiring anticoagulant treatment (low-molecular -weight -heparin 100 IU/kg × 2/24 h followed by Warfarin for 6 months. After treatment, the patient was transferred to our department of Internal Medicine for the etiological investigation.

On examination, the patient reported chronic diarrhea and weight loss with no other associated symptoms. Physical examination revealed an underweight patient (BMI:16.9) with pale conjunctiva, improvement of hemiparesis, and no other abnormalities. Laboratory tests noted biological signs of malabsorption. [(Hemoglobin: 10 g/dl (normal range > 12 g/dl), Albumin: 17.9 g/L, cholesterol: 2.8 mmol/l (normal range < 5,1 mmol/l).]

Thrombophilia assessment revealed a protein C deficiency 57% (normal range: 70-120%), a slight increase in anticardiolipin antibodies 11 IU/ml (normal range <7 UI/ml) and anti-Beta 2 glycoprotein 1 antibodies 18 IU/ml (normal range < 8 IU/ml) with normal levels of protein S, antithrombin III and homocysteinemia, and negative factor V Leiden. Immunological tests noted positive anti-transglutaminase >50 IU/ml (normal range < 8 U/ml) and anti-endomysium antibodies at 0.6 g/L (normal range < 0.2 g/L).

From examination and laboratory results, VTE was diagnosed and CD as the cause was suspected. Duodenal biopsy demonstrated villous atrophy, meaning that the diagnosis of CD could be retained after ruling out the other causes of VTE as the neoplastic aetiologies; gynaecologic examination didn’t show a lesion, neither chest radiography or colonoscopy.

The outcome of the patient was deemed favorable with anticoagulant therapy (low-molecular weight Heparin followed by Warfarin for 6 months without bleeding complications), combined with a gluten-free diet during the follow-ups over a period of 3-years in our outpatient consultation.

## Discussion

CD is defined as a chronic immune-mediated small intestinal enteropathy caused by gluten intolerance in genetically predisposed individuals. ^5^ The activation of both the innate and adaptive response of the immune system, following the ingestions of gluten leads to damage to the proximal mucosa of the small intestine, resulting in the malabsorption of nutrients and the appearance of extra-intestinal manifestations.

CD is a systemic disorder, with different forms of clinical manifestations, from a classic digestive clinical presentation to extra-intestinal symptoms. The intestinal form of CD is more commonly found in the pediatric population ^6^ and rarely in adults. It includes diarrhea, which is a common presenting sign, in addition to malabsorption symptoms.

Nevertheless, extra-intestinal manifestations are being increasingly recognized, most likely due to better awareness of atypical presentations. They can include chronic fatigue, anemia, osteoporosis, recurrent aphthous stomatitis, elevated liver enzymes, joint or muscle pain, epilepsy, peripheral neuropathy, and infertility ^7^ Therefore, it is reported that extra-intestinal manifestations may appear before the diagnosis of CD, as shown in our case.

It has been recognized that chronic inflammation is also an independent risk factor for VTE as the consequence of inflammatory cytokines and oxidative stress on the coagulation cascade is demonstrated. ^8^

Our patient presented a deficiency of protein C; which has been reported in previous studies related to CD, results in the over activity of coagulation factors V and VIII thus increasing the risk for thrombotic events. ^9^ We noted also a slight increase in anticardiolipin antibodies and anti-Beta 2 glycoprotein 1 antibodies, as shown in several studies where a higher prevalence of autoantibodies among patients with CD, including anti-phospholipid antibodies (see review of studies in ^9^). It is possible that these anti-phospholipid antibodies might also contribute to hypercoagulability.

A thrombosis assessment should be considered in patients with CD as the risk factors for thrombosis can be acquired in CD by a vitamin deficiency or antiphospholipid antibodies. These factors must be investigated, corrected, and even thromboembolic prophylaxis should be initiated.

VTE as a presentation of CD is unusual and rarely reported, especially since this thrombosis is located in the cerebrum. The seriousness of these manifestations show that malabsorption syndrome should be systematically investigated to explore any symptoms due to systemic complications of malabsorption, for early diagnosis and better prognosis. Furthermore, a long diagnostic delay may increase the risk of poor clinical response. ^10^

In fact, other central nervous system manifestations were reported more associated to CD than cerebral thrombosis, including cerebellar ataxia, peripheral neuropathy, seizures, headache, cognitive impairment, and psychiatric symptoms. ^11^

Early diagnosis and treatment of CD improves the quality-of-life for patients and may spare them various long-term or even fatal complications like thromboembolic diseases.

## Data availability

All data underlying the results are available as part of the article and no additional source data are required.

## Consent

Written informed consent for publication of the clinical details and associated images was obtained from the patient.
