# Supplementary material for: Case Report: Cerebral venous thrombosis revealing celiac disease
Source: F1000Res. 2021 Sep 10;10:680. Originally published 2021 Jul 28. [Version 2] doi: 10.12688/f1000research.54233.2 (PMC8447050; doi:10.12688/f1000research.54233.2)
Supplement: Peer review history

# Peer review: Reviewer response for version 2

**DOI:** 10.5256/f1000research.77205.r94003

**Published:** 2021-09-16

**Authors:** Bouomrani Salem

**Affiliations:** 1 Department of Internal Medicine, Military Hospital of Gabes, Gabes, Tunisia

The authors have made the requested changes. The paper in its revised version can be indexed without any further modifications.

Are enough details provided of any physical examination and diagnostic tests, treatment given and outcomes?

No

Is the case presented with sufficient detail to be useful for other practitioners?

Partly

Is sufficient discussion included of the importance of the findings and their relevance to future understanding of disease processes, diagnosis or treatment?

Partly

Is the background of the case’s history and progression described in sufficient detail?

No

Reviewer Expertise:

Autoimmune diseases, Internal Medicine, Thrombophilias

I confirm that I have read this submission and believe that I have an appropriate level of expertise to confirm that it is of an acceptable scientific standard.

# Peer review: Reviewer response for version 2

**DOI:** 10.5256/f1000research.77205.r94004

**Published:** 2021-09-13

**Authors:** Juha Taavela

**Affiliations:** 1 Celiac Disease Research Center, Faculty of Medicine and Health Technology, Tampere University, Tampere, Finland; 2 Central Finland Central Hospital, Jyväskylä, Finland

I accept the article by Wiem et al. entitled "Case Report: Cerebral venous thrombosis revealing celiac disease". The authors have made significant improvements to the intellectual content of the article and I have no further remarks.

Are enough details provided of any physical examination and diagnostic tests, treatment given and outcomes?

Yes

Is the case presented with sufficient detail to be useful for other practitioners?

Yes

Is sufficient discussion included of the importance of the findings and their relevance to future understanding of disease processes, diagnosis or treatment?

Partly

Is the background of the case’s history and progression described in sufficient detail?

Yes

Reviewer Expertise:

Celiac disease, gluten sensitivity

I confirm that I have read this submission and believe that I have an appropriate level of expertise to confirm that it is of an acceptable scientific standard.

# Peer review: Reviewer response for version 1

**DOI:** 10.5256/f1000research.57696.r91588

**Published:** 2021-08-27

**Authors:** Bouomrani Salem

**Affiliations:** 1 Department of Internal Medicine, Military Hospital of Gabes, Gabes, Tunisia

The authors present an original case report of cerebral venous thrombosis occurring in a patient with celiac disease and discuss the causal link between these two pathologies. Indeed, thromboembolic complications are part of the possible extra-intestinal manifestations of this disease with several cases and different locations reported.

 The authors insist on the fact that the revealing character, as well as the status epilepticus, is the element which makes the originality of their observation.

 Some modifications are necessary before the indexing of this manuscript:

1. Keywords: add "cerebral venous thrombosis" to the list of keywords
2. Introduction: remove the last sentence "This presentation has not been reported previously in the literature." and replace it with "this clinical situation remains exceptional and unusual during CD". Indeed, several cases of cerebral venous thrombosis as the first manifestation of CD have been reported in the literature, some of which are associated with convulsive seizures or epilepsy. Seizures are nonspecific and can simply be a consequence of cerebral thrombosis. This possibility must be mentioned.
3. Case report: the assessment of thrombophilia is incomplete. The other tests should be noted (if they were performed): prothrombin G20210A mutation (Factor II Mutation)? Tumor markers? Anti nuclear antibodies? Lupus anticoagulant?
4. Discussion:
  The causal link between CVT and CD must be well discussed and the possible mechanisms of hypercoagulability during this disease well explained i.e. deficiencies in B12, folate and vitamin K, persistent chronic inflammation, hyperhomocysteinemia, the presence of anti-phospholipids antibodies, fibrinolysis abnormalities, endothelial dysfunction, thrombocytosis, etc.
  The presence of antiphospholipid antibodies in this patient should also be discussed: simple positivity of these autoantibodies already reported during CD, or an authentic antiphospholipid syndrome associated with CD (venous thrombosis + positive anticardiolipin antibodies and anti-Beta 2 glycoprotein 1 antibodies). The association of these two autoimmune diseases remains a possible eventuality! ^1^ Is the control of these autoantibodies carried out at 12 weeks?
  The possible origin of protein C deficiency must also be discussed: a real thrombophilia? protein loss through diarrhea? or the received anticoagulant treatment (Warfarin)?
5. Bibliography: the list of bibliographic references is not well selected. Indeed, to support the discussion, the cases reporting CVT and CD, in particular the inaugural forms and associated with epilepsy, may be cited and used in the discussion: [refs 2-6]
  Likewise, the very latest update on thromboembolic and cardiovascular complications associated with CD should be cited in the list of bibliographic references and used to discuss the different possible mechanisms of thrombogenesis during this disease ^7^.

Are enough details provided of any physical examination and diagnostic tests, treatment given and outcomes?

No

Is the case presented with sufficient detail to be useful for other practitioners?

Partly

Is sufficient discussion included of the importance of the findings and their relevance to future understanding of disease processes, diagnosis or treatment?

Partly

Is the background of the case’s history and progression described in sufficient detail?

No

Reviewer Expertise:

Autoimmune diseases, Internal Medicine, Thrombophilias

I confirm that I have read this submission and believe that I have an appropriate level of expertise to confirm that it is of an acceptable scientific standard, however I have significant reservations, as outlined above.

## References

[1] : [Celiac disease associated with antiphospholipid syndrome].*Rev Esp Enferm Dig*.2008;100(2) : 10.4321/s1130-01082008000200009102-310.4321/s1130-0108200800020000918366270

[2] : [Celiac disease, cerebral venous thrombosis and protein S deficiency, a fortuitous association?].*J Mal Vasc*.2005;30(4 Pt 1) : 10.1016/s0398-0499(05)88207-1228-3010.1016/s0398-0499(05)88207-116292200

[3] : Concurrent cerebral arterial and venous sinus thrombosis revealing celiac disease- a case report and literature review.*BMC Gastroenterol*.2020;20(1) : 10.1186/s12876-020-01483-w32710.1186/s12876-020-01483-w33023506PMC7542124

[4] : Recurrent cerebral venous thrombosis revealing celiac disease: an exceptional case report.*Acta Neurol Belg*.117(1) : 10.1007/s13760-016-0698-y341-34310.1007/s13760-016-0698-y27680734

[5] : [Association of celiac disease and cerebral venous thrombosis: report of two cases].*J Mal Vasc*.2013;38(1) : 10.1016/j.jmv.2012.11.00347-5110.1016/j.jmv.2012.11.00323287617

[6] : Cerebral venous sinus thrombosis in 2 children with celiac disease.*Clin Appl Thromb Hemost*.2011;17(5) : 10.1177/1076029610369799466-910.1177/107602961036979920530061

[7] : Thromboembolic complications and cardiovascular events associated with celiac disease.*Ir J Med Sci*.2021;190(1) : 10.1007/s11845-020-02315-2133-14110.1007/s11845-020-02315-232691305

# Peer review: Reviewer response for version 1

**DOI:** 10.5256/f1000research.57696.r92258

**Published:** 2021-08-23

**Authors:** Juha Taavela

**Affiliations:** 1 Celiac Disease Research Center, Faculty of Medicine and Health Technology, Tampere University, Tampere, Finland; 2 Central Finland Central Hospital, Jyväskylä, Finland

The authors have found celiac disease in a patient with venous thromboembolism. I especially like the conclusion that early diagnosis of CD is needed which is an important topic in CD (Popp *et al.*, 2019). The case report is interesting, however, I have some comments:

- The link between celiac disease and VTE is unknown. The authors suggest in the discussion that a thrombosis assessment should be considered in CD patients. Such a conclusion cannot be drawn from a couple of case reports. And the suggestion of thromboembolic prophylaxis is also not supported by such small data. I believe the situation is similar as in IBD, in which the acute phase causes a risk for VTE (Grainge *et al*, 2010). However, celiac disease is a much easier disease to treat than some IBDs, and such acute situations in the ER are not seen in CD. This must be changed in the discussion.
- Another thing to discuss is that is the cause of protein C deficiency, coagulation factors and anti-phospholipid antibodies CD or just the fulminant diarrhea and nutrition deficiency?
- As a minor remark the article should also mention that a significant proportion of celiacs are nowadays found while screening in-at risk groups such as Graves disease, type 1 diabetes etc (2). And these patients are without any symptoms but they benefit from treatment (Kurppa *et al., *2014).

Are enough details provided of any physical examination and diagnostic tests, treatment given and outcomes?

Yes

Is the case presented with sufficient detail to be useful for other practitioners?

Yes

Is sufficient discussion included of the importance of the findings and their relevance to future understanding of disease processes, diagnosis or treatment?

Partly

Is the background of the case’s history and progression described in sufficient detail?

Yes

Reviewer Expertise:

Celiac disease, gluten sensitivity

I confirm that I have read this submission and believe that I have an appropriate level of expertise to confirm that it is of an acceptable scientific standard, however I have significant reservations, as outlined above.

## References

[1] : Venous thromboembolism during active disease and remission in inflammatory bowel disease: a cohort study. *The Lancet*.2010;375(9715) : 10.1016/S0140-6736(09)61963-2657-66310.1016/S0140-6736(09)61963-220149425

[2] : Diagnosing Celiac Disease: Towards Wide-Scale Screening and Serology-Based Criteria?. *Gastroenterol Res Pract*.2019;2019: 10.1155/2019/2916024291602410.1155/2019/291602431467522PMC6701393

[3] : Benefits of a gluten-free diet for asymptomatic patients with serologic markers of celiac disease.*Gastroenterology*.2014;147(3) : 10.1053/j.gastro.2014.05.003610-617.e110.1053/j.gastro.2014.05.00324837306
